# Outbreak of dengue fever in Ghana: The emergence of DENV-1 serotype

**DOI:** 10.1371/journal.pntd.0014248

**Published:** 2026-05-11

**Authors:** Deborah Pratt, Yaw Awuku Larbi, Magdalene Ofori, Bright Agbodzi, Jessica Wiley, Ama O. A. Mante, Selassie Kumordjie, Miriam Eshun, Stella Bour, Nancy Enimil, Juliana N. D. Acquah-Amaning, Prince Ketorwoley, Maame S. Boapea, David D. Nutakor, Musah Salisu, Gertrude Stephens, Emmanuel A. Boateng, Tracy Adjandeh, Joel Koomson, Dennis Laryea, Franklin Asiedu-Bekoe, Patrick Kuma-Aboagye, Patrick Mawupemor Avevor, Argata G. Guracha, Sally-Ann Ohene, Terrel Sanders, Michael Wiley, Joseph Humphrey Kofi Bonney

**Affiliations:** 1 Virology Department, Noguchi Memorial Institute for Medical Research, University of Ghana, Legon, Ghana; 2 Naval Medical Unit EURAFCENT, Accra, Ghana; 3 University of Florida Genetics Institute, Gainesville, Florida, United States of America; 4 PathoSeq Bio LLC, Omaha, Nebraska, United States of America; 5 Parasitology Department, Noguchi Memorial Institute for Medical Research, University of Ghana, Legon, Ghana; 6 Public Health Division, Ghana Health Service, Ministry of Health, Accra, Ghana; 7 Country Office Accra, World Health Organization, Accra, Ghana; 8 Department of Pathology, Microbiology and Immunology, College of Medicine, University of Nebraska Medical Centre, Omaha, Nebraska, United States of America; Faculty of Science, Ain Shams University (ASU), EGYPT

## Abstract

**Background:**

Dengue, a mosquito-borne virus continues to be a public health concern in the tropics and subtropical parts of Africa. Due to the increase of urbanization, climate change and trans-Atlantic trade, the transmitting vector, Aedes aegypti, has become prevalent hence the rapid growth of the disease, globally. In Ghana, there have been sporadic laboratory-confirmed cases of dengue reported over the years through surveillance activities. However, despite the detection of these cases, Ghana had never experienced a major outbreak (unlike its neighboring countries) until July 2024.

**Methods:**

During this outbreak, a total of 1471 suspected dengue fever specimen received from various health facilities in Ghana in NMIMR for molecular diagnostic testing using a RT-qPCR assay for dengue, chikungunya and Zika viruses and selected positives sequenced using Illumina Next Generation Sequencing.

**Results:**

Dengue fever virus RNA was detected from 206 samples and serotyped as DENV-1 with one DENV-3 coinfection. Thirty-nine genomes were successfully generated after sequencing and phylogenetic analysis of DENV-1 strains revealed two main clusters with AFI isolates in Ghana and isolates from other West African countries (Côte d’Ivoire, Burkina Faso, Benin, and Senegal) circulating between 2017–2019. In 2023, DENV-1 was frequently isolated which could account for it being the predominant serotype transmitted in the recent outbreak.

**Conclusion:**

The outbreak response and the case management procedures deployed by the health authorities during this outbreak were swift and was enough to prevent a fatal difficult-to-control situation. With the absence of a widely accepted commercialized vaccine and treatment for dengue fever, there is a need to enhance surveillance activities and control the vectors which can transmit DENV in-country to curb the occurrence of outbreaks.

## Author summary

Dengue fever is a febrile illness that occurs yearly in most part of Africa. This disease is usually asymptomatic but can progress to severe disease and even death. Prior to this major outbreak, in 2022, Ghana had started recording cases of DENV 1 even though DENV 2 and 3 were the known serotypes circulating. The sequences of DENV 1 obtained had similarities with the strains circulating in the endemic African regions and that of Asia. Its presence and subsequent predominance in Ghana may partly be attributable to trans-Atlantic trade or importation of the virus. At the time of Ghana’s dengue fever outbreak, other neighbouring countries in the subregion were also experiencing this epidemic. The sequential multi-country outbreaks in the sub region at the time presented a source of concern albeit, timely and careful management of the crisis averted a major fatal outbreak. Though the spread was minimized and controlled, there is a need to improve and strengthen the regional emergency response and preparedness measures for outbreaks in general and dengue fever in particular. The dengue fever outbreak in Ghana however highlights the essence of the surveillance system built in the country and the relevance of early detection which helped to curb the upsurge of the disease.

## Introduction

Post-pandemic era, there has been an increase in the global burden of dengue fever (DF) according to alarming statistical reports by the World Health Organization (WHO) [1]. Earlier in the year, as of July 23, the WHO reported over 10 million dengue cases in 176 countries with over 24,000 severe cases and 6,508 deaths [2]. By the end of 2024, the global surveillance for dengue by WHO reported ~13.8 million dengue cases with 9,965 total deaths The global surge in dengue cases portrays its growing threat as a public health crisis.

The global burden of dengue can be attributable to several factors including urbanization, increased international travel [3], and climate change [4]. These factors have been associated with dengue outbreaks and may have aided active transmission of the virus through its mosquito vector. It has also been established that climate change is likely to expand geographical distribution of mosquito-borne diseases [5]. Additionally, El Niño events have been linked to periodic outbreaks of mosquito-borne diseases like malaria and dengue by altering rainfall and temperature patterns that favour mosquito proliferation [6,7]. This has raised concerns about its potential effects on the transmission of infectious diseases, particularly dengue [8].

Dengue fever is an arthropod-borne viral infection caused by the dengue virus (DENV). It is a member of the family *Orthoflavivirus*, with 4 antigenically distinct serotypes (DENV-1 to DENV-4) [9,10]. The virus is transmitted by the *Aedes* mosquito and an infection presents as a mild febrile illness but can progress into severe Dengue Hemorrhagic Fever (DHS)/Dengue Shock Syndrome (DSS) [11]. An infection with one serotype does not confer protective lifelong immunity against another serotype but rather increases the risk of severe dengue [12]. In Africa, all four DENV serotypes have been isolated with DENV-2 frequently reported in most epidemics [13]. Between 1960 and 2020, out of 45 outbreaks recorded, 17 and 16 occurred in East (mainly Kenya, Eritrea) and West Africa (mainly in Burkina Faso, Senegal, Cote d’Ivoire), respectively, with DENV-1 and DENV-2 as the predominant serotypes contributing to 60% of the epidemics [14].

Reports of increased dengue fever outbreaks in recent years suggest active and potentially expanding transmission of the virus throughout Africa [15]. While this rise may partly reflect improved diagnostic capacity, surveillance activities and heightened awareness of dengue [16–19], the factors driving the virus transmission such as expansion of mosquito habitats, urbanization, and inadequate vector control measures still remain. Despite these developments, the African continent faces significant challenges including funding constraints which hinder preparedness and response to dengue outbreaks [20,21]. Another example is the limited clinical experience by health staff in disease diagnosis, with many primary infections going undetected or misdiagnosed as malaria [22–24].

In West Africa, surveillance of arboviral diseases including dengue has historically been sparse. However, various West African countries have experienced multiple arboviral outbreaks including those caused by dengue virus with notable ones reported particularly in Burkina Faso and Côte d’Ivoire [25,26]. Although the countries surrounding Ghana had experienced epidemics of dengue fever over the years, Ghana until recently had documented only serological evidence of dengue virus without any confirmed case [18,27] suggesting low-level circulation or the lack of early detection of the virus despite the abundance of *Ae. Aegypti* mosquitoes in Ghana [28]. This changed in 2017 when dengue virus was detected in children with suspected malaria using a multi-pathogen PCR assay [17]. Subsequently, Ghana reported the presence of two serotypes (DENV-2 and DENV-3) in the sera of clinically suspected Ebola Virus Disease (EVD) patients during the EVD outbreak in West Africa [29]. Since then, sporadic cases of dengue fever have been reported in the country, but no major outbreak documented [30].

Recently, (between 2022 – 2023) DENV-1 was frequently isolated in-country, increasing the number of circulating dengue virus serotypes in Ghana to three [30]. Again, in June 2024, another isolated case which was serotyped into DENV-3 [26] was detected in the Volta region. Then this reported full-scale outbreak started a month later, from July 2024 with hotspots identified in the eastern and central regions. In this paper, we report on the first major outbreak of dengue fever in Ghana marked by the emergence of DENV-1 serotype.

## Methods

### Study site, case definition and clinical specimen

As a response to hemorrhagic fever outbreaks in neighboring countries in West Africa, a national surveillance system was set up in 2014 in Ghana as part of an EVD preparedness and response plan to detect and rapidly respond to cases and rumors of cases. The Ghana Health Service and World Health Organization have designated the Noguchi Memorial Institute for Medical Research (NMIMR) as a reference laboratory for the investigation of Dengue and other hemorrhagic fever viruses such as Lassa, Ebola & Marburg from suspected cases of dengue fever or acute hemorrhagic fever (AHF) syndrome [29]. The Ghana Integrated Disease Surveillance Response Strategy (GIDSR) recognizes both AHF & Dengue Fever as epidemic prone diseases requiring immediate reporting. Healthcare facilities including regional, municipal, district, polyclinics and health centres under the Ghana Health Service across the 16 regions in-country submit clinical specimen of serum/plasma from patients suspected of VHFs to the NMIMR. The clinical specimens are submitted together with case investigation forms containing the demographic and clinical data of the patients. A suspected case for dengue fever was defined per the Integrated Disease Surveillance and Response Strategy (IDSR) in Ghana [31], as any person with acute febrile illness of 2–7 days duration with 2 or more of the following: headache, retro-orbital pain, myalgia, arthralgia, rash, hemorrhagic manifestations, leucopaenia. A confirmed case was defined as a suspected case with laboratory confirmation (positive IgM antibody, fourfold or greater increase in IgG antibody titres in paired (acute and convalescent) serum specimens, positive PCR) [32].

### Laboratory testing

Clinical blood specimens of patients suspected of VHFs or acute febrile illness were brought to NMIMR for molecular diagnostic testing. All samples were processed in a Biosafety Level 2 (BSL-2) containment facility.

### Nucleic acid extraction

Blood samples placed in EDTA collection tubes or serum separator tubes were centrifuged for 10 minutes at 1500 rpm using the Kubota centrifuge to obtain serum or plasma. Viral RNA was extracted from 140 μL of the clinical specimen using the QIAamp Viral RNA kit (Qiagen, Hilden, Germany) following the manufacturer’s instructions. The viral nucleic acid was eluted at 60 µL.

### Real time reverse transcription quantitative polymerase chain reaction (rRT-qPCR)

Polymerase chain reaction was performed using a CDC Trioplex Real-time RT-PCR Assay for detection of dengue, chikungunya, and Zika viruses with an Invitrogen SuperScript III One-Step RT-PCR System with PlatinumTaq High Fidelity DNA Polymerase.

Samples testing positive for dengue virus were further serotyped using a TaqMan-based real-time reverse transcription-polymerase chain reaction (rRT-PCR) assay established by Johnson et al., 2005 classify the positives into the four DENV serotypes. All PCR amplification processed were performed using the Applied Biosystems 7500 Fast Real-time PCR instrument.

### Whole genome sequencing

Sequenced libraries were prepared using the Illumina DNA prep with enrichment kit (Illumina Inc, San Diego, CA, USA) according to manufacturer’s instructions, with slight modifications. A cDNA NF-ONT primer mix (Pathoseq Bio, LLC Omaha, USA) was used at the adapter amplification step. Viral enrichment was performed using custom target probes, twist viral probe version II (Twist biosciences, San Francisco, CA, USA). Extracted RNA was reverse transcribed to cDNA and libraries barcoded and sequenced on the MinION using the FLO-MIN10 V10 flowcell (Oxford Nanopore Technologies plc, Oxford, UK).

### Phylogenetic analysis

Genotyping for DENV isolates was conducted using the Flavivirus Virus Genotyping Tool available online at https://www.rivm.nl/mpf/typingtool/flavivirus/. To construct the phylogeny for DENV-1 isolates, BLAST searches were performed on individual genomes from the outbreak, selecting the top ten matching sequences for each query. In addition, all available genomes of African origin on GenBank we could locate as of September 20, 2024, were included. To ensure broad geographical and temporal representation, genomes from major continents, including the Americas, Asia, and Oceania, spanning 1944–2021, were included, resulting in a dataset of 334 genomes. The sequences were aligned using MAFFT v7.526 (https://mafft.cbrc.jp/alignment/software/). To address ambiguities caused by polymorphisms in the 5′ and 3′ untranslated regions, sequences were trimmed to the open reading frames (ORFs), which were used for all subsequent phylogenetic analyses.

Recombination analysis was conducted using RDP5 software (Martin et al., 2021), utilizing multiple algorithms such as RDP, GENECONV, BootScan, MaxChi, and SiScan. Recombination events were confirmed if signals were detected by at least two algorithms with p-values < 0.05. Fourteen recombinant sequences were removed to create a recombination-free dataset of 334 genomes for further analysis. A maximum likelihood (ML) phylogeny was constructed in IQ-TREE v2.0.3 (Minh et al., 2020) using 1000 ultrafast bootstraps. The GTR + I + G model was identified as the best-fit nucleotide substitution model. Bayesian inference was performed with BEAST v1.10.4 to investigate the evolutionary dynamics and estimate the time to the most recent common ancestor (TMRCA) of the studied strains. ORFs aligned in MAFFT served as input for BEAST analyses. Temporal signal assessment was performed through root-to-tip genetic divergence and sampling date correlation analysis using TempEst v1.5.3 (http://tree.bio.ed.ac.uk/software/tempest/). The Bayesian analysis employed a strict molecular clock and the Bayesian Skyride coalescent model, selected based on marginal likelihood estimates obtained via path sampling and stepping-stone sampling. Fifty million MCMC steps were performed on five independent chains using the GTR + I + G substitution model. Convergence of parameters was assessed in TRACER v1.7.1 to ensure an effective sample size (ESS) ≥ 200. Tree files were merged using LogCombiner v1.10.4, and a maximum clade credibility (MCC) tree was generated with TreeAnnotator, using 10% of the dataset as burn-in. Tree visualization and annotation were conducted in FigTree v1.4.4 and Inkscape v1.2. For DENV-3, BLAST searches were performed using outbreak genomes, selecting the top matching sequences. Additionally, all African-origin genomes available on GenBank as of September 30, 2024, were included. The dataset was processed similarly to DENV-1, except molecular clock analyses were not performed.

### Statistical analysis

Patients’ characteristics and symptoms by dengue fever detections were presented as percentages and frequencies using Microsoft Excel. The positivity rates of dengue (percentage of clinical specimen received that tested positive for the presence of the dengue virus) were described by epidemiological weeks and presented as an epi-curve using Microsoft Excel. The Chi Squared Test was used to determine associations between patient characteristics and dengue detections. Associations obtained from the Chi-Squared Test with p-value <0.05 were considered significant. The Wald’s test was used to compute the limit of confidence intervals in STATA v16. The maps generated using QGIS v3.26.1-Buenos Aires with the boundary coordinates obtained from Ghana Statistical Service (GSS).

## Results

### Overall

Between January 15 and September 31, 2024, the NMIMR processed samples collected from 1471 individuals suspected of dengue virus infection out of which 206 (14.0%) tested positive for the virus. All samples tested negative for chikungunya and Zika Viruses. We serotyped 84 out of the 206 dengue positive cases due to inadequate serotyping reagents. A total of 81 out of the 84 serotyped dengue positive samples were DENV-1 serotype only, 2 were DENV-3 serotype only and 1 case had coinfection of DENV-1 & DENV-3.

### Demographic characteristics of dengue cases

The median age among all individuals whose samples were processed was 32 years (interquartile range: 19–46 years) among the dengue cases was 36 years (interquartile range: 25–49 years). The demographic characteristics of the samples analyzed are shown in Table 1. Dengue prevalence was highest among individuals aged 50–59 years (18.2%, Percentage Confidence Interval (PCI): 13.0%-25.0%) and lowest among individuals aged <10 years (8.0%, Percentage Confidence Interval (PCI): 4.0%-13.1%) (Table 1). We detected dengue in more females 15.1% (PCI: 12.8% - 17.7%) than in males 12.7% (PCI: 10.4% - 15.5%).

**Table 1:** Demographic characteristics of dengue positive cases in the outbreak.

Characteristics	Total, N	Positive, n	Percentage^a^, % (CI)	p-value
**Age in years, median (IQR)**	32 (19–46)	36 (25–49)		
**Age group**				
**< 10**	175	14	8.0 (4.8–13.1)	0.055
**10–19**	205	22	10.7 (7.2–15.8)	
**20–29**	263	35	13.3 (9.7–18.0)	
**30–39**	317	49	15.5 (11.9–19.9)	
**40–49**	198	35	17.5 (13.0–23.6)	
**50–59**	159	29	18.2 (13.0–25.0)	
**60+**	143	21	14.7 (9.8–21.5)	
**N/A**	11	1	9.0 (0.2–41.3)	
**Status**				
**Alive**	1419	203	14.3 (12.6–16.2)	0.869
**Died**	6	1	16.7 (2.3–63.2)	
**N/A**	46	2	4.3 (0.5–14.8)	
**Gender**				
**Female**	802	121	15.1 (12.8–17.7)	0.198
**Male**	667	85	12.7 (10.4–15.5)	
**N/A**	2	0	–	
**Overall**	1471	206	14.0 (12.3–15.9)	

^a^% prevalence is the n/N and the CI is the confidence intervals of the percentage estimates.

### Geographical distribution of dengue cases

**Fig 1:**
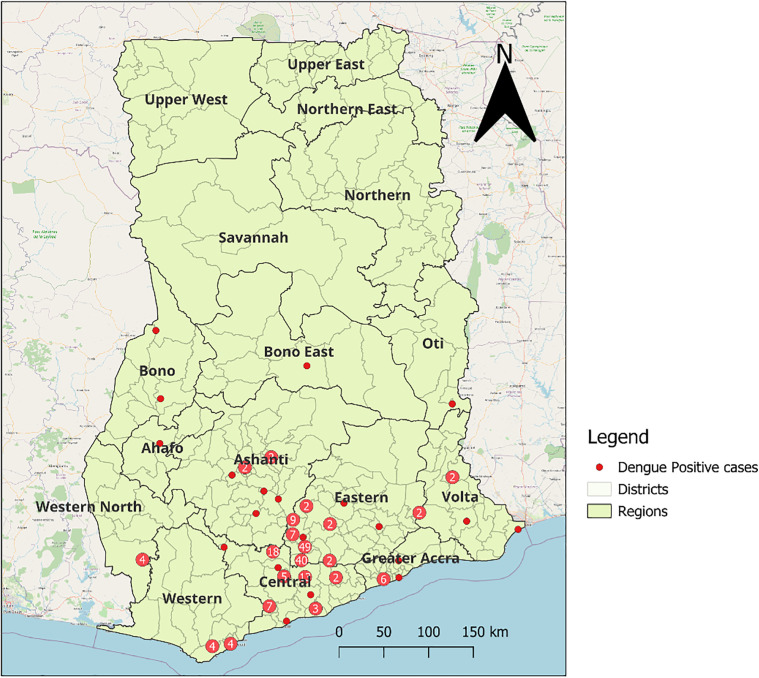
The distribution of recorded dengue cases in Ghana from January to September, 2024. The map was created using QGIS v3.26.1-Buenos Aires. The administrative boundaries coordinates obtained from Ghana COD-AB (Humanitarian Data Exchange, https://data.humdata.org/dataset/cod-ab-gha), CC BY 4.0. Basemap: OpenStreetMap “Standard” (OpenStreetMap contributors; data under ODbL 1.0, https://www.openstreetmap.org/copyright). The red dots represent a single DENV positive within the districts and regions of Ghana. A red dot with a number in the middle symbolized the number dengue cases detected with that area.

During the reporting period, dengue cases were detected in 11 out of the 16 regions. Most of the cases were concentrated in the southern part of Ghana, especially in the Eastern and Central Regions, with no reported cases in the northern part of Ghana.. Dengue was also detected in other regions as shown in Fig 1.

### Temporal distribution of dengue cases

**Fig 2:**
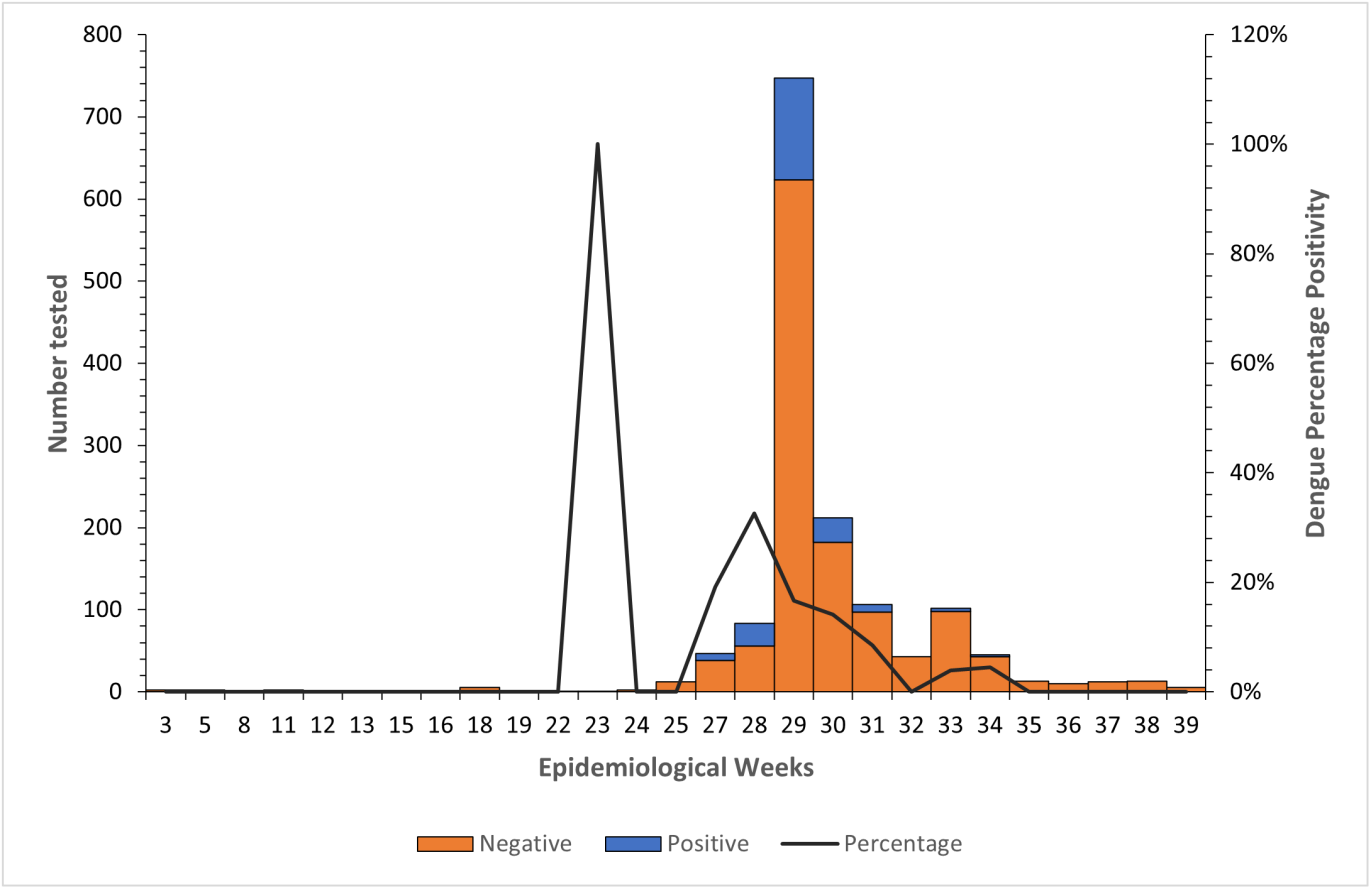
Distribution of dengue cases by epidemiological weeks from January to September 2024. This graph was generated using Microsoft Excel 2016. Weeks are shown in WHO calendar years (ISO-8601). Numbers tested across the weeks are represented as bars with dengue positives in blue stacked over dengue negatives in orange and dengue percentage positivity rates are traced with lines.

Dengue detections occurred from 03 June to 21 August 2024 (Epidemiological weeks 23–34) (S1 Fig). The first dengue case in the year was detected on 03 June 2024 (Epidemiological week 23). From week 27 (01–07 July) we observed a rapid increase in number of DENV positives until week 28 (08–14 July) when the percentage positivity peaked (33%) (Fig 2). However, in terms of numbers, 121 cases were detected in week 29 (15–24 July). By week 29 and 30, the cases had spread from the hotspots to other communities in neighbouring regions (S1 Fig). The number of dengue cases began to decline with a few cases occurring between Epi week 33 and 35.

### Clinical manifestations of dengue cases

All confirmed cases reported at least one symptom. Headache was the most common condition (92.8%) among the cases, followed by fever (84%) and muscle joint pain (73.5%). The symptom profile of the dengue cases is shown in Table 2.

**Table 2:** Clinical manifestation of dengue cases.

Clinical Manifestations	Dengue cases	
	%	(n/N)^a^
Headache	92.8	180/194
Fever	84	158/188
Muscle Joint pain	73.5	133/181
Anorexia	65.7	117/178
Intense fatigue	62.9	112/178
Vomiting/nausea	53.7	95/177
Abdominal Pain	47.4	81/171
Diarrhoea	36.5	62/170
Difficulty swallowing	13.3	22/166
Difficulty breathing	11.3	19/168
Bleeding	9.3	14/151
Skin rash	5.3	8/150
Hiccoughs	3.4	5/148

^a^n represents number of dengue cases with symptom present, N represents number of patients reporting symptoms.

### Sequencing analysis

**Fig 3:**
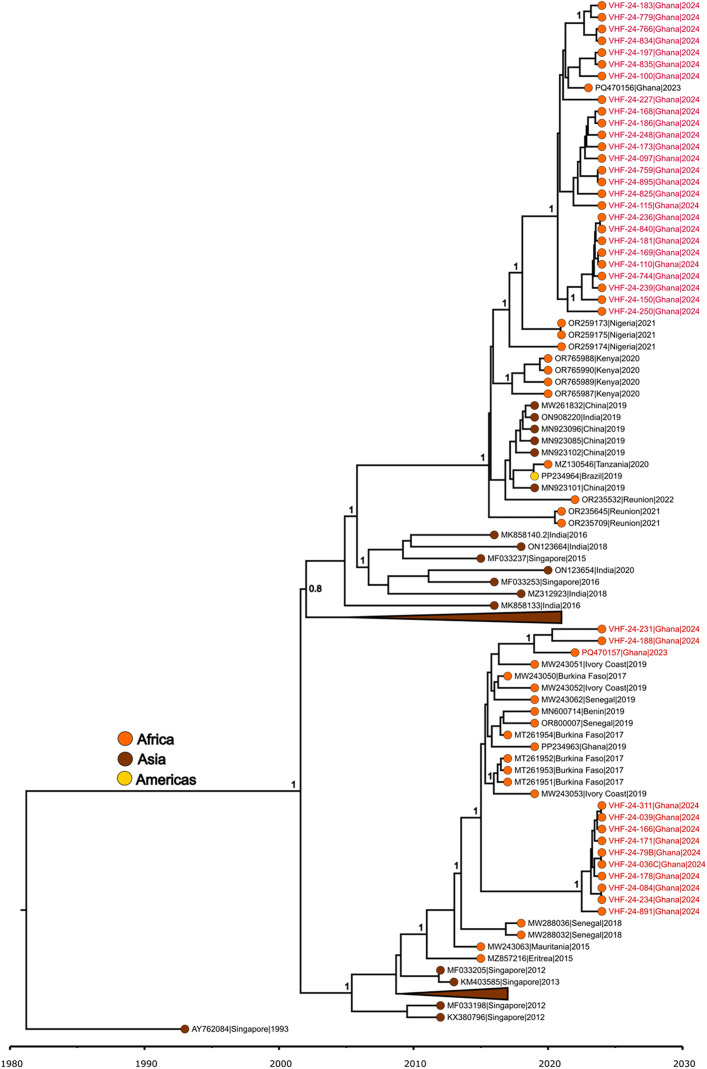
Time-calibrated phylogeny of a subset of global dengue 1 virus genomes alongside the Ghana 2024 outbreak strains (red). Colored circles indicate geographic origins. Posterior probabilities are shown at major nodes. Taxon labels include accession numbers, countries, and years of isolation for sequences extracted from GenBank, while new sequences are labeled with strain names.

Sequencing analysis from the positives generated 38 and one coding complete DENV-1 DENV-3 genomes respectively. Genotyping analysis classified all DENV-1 and DENV-3 genomes as genotype III. Phylogenetic analysis of DENV-1 strains revealed two main clusters (Fig 3). **Cluster 1** formed a monophyletic clade comprising 26 isolates from the current outbreak and a 2023 isolate from Ghana (PQ470156.1) reported during acute febrile illness (AFI) surveillance. This clade was closely related to 2021 isolates that cryptically circulated in Nigeria. **Cluster 2** was divided into two subclusters. *Subcluster 1* included 2 isolates from the current outbreak, one 2022 Ghana isolate reported during AFI surveillance, and isolates from West African countries such as Côte d’Ivoire, Burkina Faso, Benin, and Senegal, circulating between 2017 and 2019. *Subcluster 2* was a monophyletic clade of 10 isolates from the current outbreak, closely related to 2018 isolates from Senegal (Fig 3).

For molecular clock analysis, regression of root-to-tip genetic distances against sampling dates yielded a correlation coefficient of 0.94 and an R-squared value of 0.88, indicating a strong temporal signal in the dataset. The time-calibrated phylogeny estimated the TMRCA of Cluster 1 to be around 2021 (95% highest posterior density [HPD]: 2020.1–2021.2). The GH-Asia-Africa clade was estimated to have a TMRCA around mid-2015 (95% HPD: 2014.9–2016.3). The TMRCA of Cluster 2 was dated to early 2015 (95% HPD: 2014.8–2015.8), with Subcluster 2 having a TMRCA around mid-2022 (95% HPD: 2021.9–2023). The mean evolutionary clock rate for the dataset was 5.23 × 10 ⁻ ⁴ substitutions/site/year (95% HPD: 5.0 × 10 ⁻ ⁴–5.5 × 10 ⁻ ⁴).

**Fig 4:**
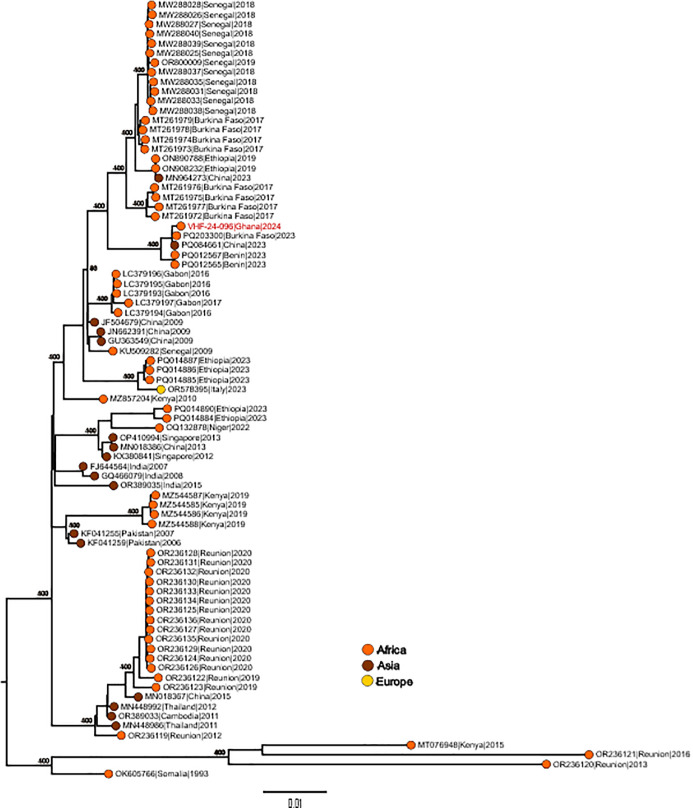
Molecular phylogenetic analysis a subset of global dengue 3 virus genomes alongside the Ghana 2024 outbreak strain (red). Tree model inference and phylogeny were simultaneously conducted in IQ-TREE v1.6.1, executing 1000 bootstrap replicates. Tip colors indicate geographical origins and are interpreted in the color key. Critical nodes are labeled with bootstrap values. The tree was visualized in FigTree.

The DENV-3 isolate from this study formed a monophyletic clade with 2023 isolates from Benin, Burkina Faso, and China (Fig 4).

## Discussion

Although, there have been sporadic cases recorded over the years, our findings indicate the first major dengue fever outbreak recorded in Ghana mainly driven by the DENV-1. In July 2024, the Ministry of Health (MoH) announced dengue fever outbreak in the Eastern region of Ghana following nine confirmed cases after laboratory investigation at the NMIMR. This increased public awareness and surveillance activities in the community, district and regional levels for early case detection and action. Post COVID-19 pandemic, neighbouring countries Cote d’Ivoire and Burkina Faso have frequently reported a number of outbreaks [1]. In early March 2023, a dengue fever outbreak in Burkina Faso involving multiple circulating serotypes signaled the potential cross-country spread of the virus [33,34]. Our findings in addition to published data from other West Africa countries suggest the ongoing transmission of DENV within the region. This highlights the need for enhanced and continuous surveillance and data sharing on dengue fever virus in Ghana, as well as awareness creation in the event of unusual detections within the country and in the region.

Between 2016 and 2018, Ghana recorded high dengue seropositivity rates especially in the southern regions such as Greater Accra [27]. The overall pooled estimate of dengue in Ghana is 33% which is substantial [35]. Over the years, surveillance activities in the country have facilitated the detection of DENV-2 and DENV-3 which circulated sporadically at different times between 2017 and 2022 [17,29,30]. Notably, DENV-1 emerged in 2022–2033 increasing the number of circulating serotypes to three within the country which is alarming [30].

Before announcement of this outbreak, in-country surveillance activities were still ongoing with no active cases reported until the detection of an isolated dengue case in June 2024 in the Volta region, which was serotyped into DENV-3 [26]. This was followed by a full-scale outbreak a month later during the minor rainy season (July to August) with hotspots identified in the eastern and central regions. The cause of this major outbreak is currently not known but it is speculated that bush/forest clearing for urbanization projects in the communities led to an upsurge of the population of the mosquito vectors from their natural habitats into households. This resulted in increased insect bites from potentially dengue infected mosquitoes and subsequent febrile cases which were confirmed later as dengue fever viral infections. During this outbreak period, environmental conditions (rainy season) supported mosquito breeding and survival [36]. The vector *Aedes* has been associated to be more rain dependent and seasonal in terms of abundance [37]. The high number of these vectors in the country has been linked to the presence of water-holding containers near households, which become mosquito breeding grounds [38]. Transportation activities may have also aided the spread of the virus to neighboring regions from the high incidence areas. Research has also demonstrated that DENV can be transported over distances exceeding 500 km through DENV-infected eggs and larvae transported in containers or by infected individuals [39] explaining the probable spread of the virus to neighboring regions.

Data analysis after laboratory investigations showed that dengue fever was detected in all age groups (≤10 – 60 + years). However, the pathogen was predominant among individuals aged 30–59 years and commonly among more females than males. This observation may be due to a combination of factors. The higher proportion of female positives could partly reflect greater vector exposure among women who were predominantly at home during the day when the bush clearing occurred. This observation may also have been influenced by higher health-seeking behaviours among women compared to men, which may have affected detection rates. While some evidence from Ghana suggests that women are slightly more likely to present for healthcare, (particularly in certain age or socioeconomic groups) this is not a universal pattern [40]. In the affected rural communities, daily activity patterns may differ between men and women. Men from these age groups may spend much of the day away from home for farming/hunting activities and the women are more likely to spend time in and around the household performing domestic chores. Since *Aedes* mosquitoes feed during the day [41], individuals who remained near households closer to the bush/forest clearing may have had greater exposure to suffer from the bites from these DENV infected *Aedes* spp mosquito vectors.

The recent outbreak was also noticeably characterized by fever, headache, muscle and joint pain with intense cases as well as diarrhoea and bleeding in some cases. These presentations were fairly similar to clinical manifestations implicated in outbreaks in other regions [42–44]. Limited information is available regarding whether the recorded death involved pre-existing health conditions. Further investigation is needed to determine if the patient had any underlying health conditions.

It is worth noting that this outbreak included a case of coinfection and cocirculation of DENV-1 and DENV-3. Cases of coinfection are more typical in endemic regions where multiple serotypes are circulating [45,46]. According to Bonney et al., 2024, there are three different serotypes of dengue circulating in the country (DENV-1, DENV-2 and DENV-3). Previous studies suggest that this could occur when a single mosquito acquires different serotypes during feeding and subsequently transmit them to humans or when two different mosquitoes carrying different serotypes infect an individual within a short time period [47]. Moreso, the circulation of the multiple serotypes among the people in the communities put them at risk of antibody-dependent enhancement (ADE) to dengue fever [48]. The majority of co-infections occur when travelers returning from dengue-endemic regions introduce the virus into their home countries [49]. The establishment of new serotypes within these regions can become permanent, depending on factors such as vector competence and the prevailing environmental conditions [45].

For the first time after sequencing analysis, we report on multiple genomes from this major outbreak. We observed circulation of DENV-1 and DENV-3 with DENV-1 being predominant. The predominance of DENV-1 in this outbreak was as a result of the spread of the virus from the central point of the outbreak (Eastern and Central regions) to nearby regions. RNA viruses have the potential to undergo spontaneous mutations [50] and these changes can occur as they infect different hosts. This highlights the importance of genomic surveillance. The key component of phylogenomics is projecting modifications/alterations in viral genomes collected from reservoirs and new hosts during and/or between epidemics onto phylogenetic trees of viruses [51]. Interestingly, phylogenetic analysis from this study revealed that DENV-1 are closely related to strains from Cote D’Ivoire, Nigeria and Burkina Faso, Benin and Senegal and for DENV-3, Cote D’Ivoire and Burkina Faso. Although we do not fully understand the transmission dynamics facilitating the virus spread, we speculate that the infection was imported from neighbouring countries, and this calls for further investigation.

Phylogenetic analysis revealed three main clusters of DENV-1 among the outbreak strains. Cluster 1, a monophyletic clade of Ghanaian isolates, shared a common ancestor in 2021, with its closest relatives being Nigerian strains reported in 2021. The Ghanaian and Nigerian strains shared a common ancestor around 2018, suggesting cryptic circulation of these strains in the region. This analysis further suggests that Ghanaian and Nigerian strains, along with other African strains, are the result of a recent introduction event from Asia around 2015. Cluster 2 of Ghanaian isolates was divided into two sub-clusters. Sub-cluster 1 included Ghanaian isolates that grouped with West African isolates sharing a common ancestor around 2015. This cluster represents an endemic lineage of West African isolates reported in the region between 2015 and 2019. Sub-cluster 2 formed a monophyletic clade of Ghanaian isolates that diverged from Sub-cluster 1 in 2015, indicating an emerging lineage endemic to Ghana. This cluster shared a common ancestor with 2018 isolates from Senegal around 2013, although the African isolates in this cluster appear to have originated from an introduction event from Asia around 2009. The DENV-3 isolate from this study formed a monophyletic clade with 2023 isolates from Benin, Burkina Faso, and China. This represents an emerging clade distinct from the endemic West African isolates.

The outbreak response and case management procedures deployed by the health authorities during this outbreak were swift and enough to prevent a fatal difficult-to-control situation. Some vector control measures were put in place to eliminate factors that support mosquito breeding and survival. This included spraying the communities and households with insecticides and general cleaning such as weeding around households and emptying cans and containers containing stagnant waters.

## Conclusion

Dengue fever continues to remain a public health threat in the tropical and subtropical regions globally. The response activities from the Ghana health service and the timely report of results to the health facilities informed adequate health interventions stopped the outbreak from exacerbating.

## Supporting information

S1 Fig Geographical distribution of cases by epidemiological weeks from the onset of the outbreak in week 27 to the end in week 34. The map was created using QGIS v3.26.1-Buenos Aires. The administrative boundaries coordinates obtained from Ghana COD-AB (Humanitarian Data Exchange, https://data.humdata.org/dataset/cod-ab-gha), CC BY 4.0. Basemap: OpenStreetMap “Standard” (OpenStreetMap contributors; data under ODbL 1.0, https://www.openstreetmap.org/copyright). The red dots represent a single DENV positive within the districts and regions of Ghana. A red dot with a number in the middle symbolized the number dengue cases detected with that area. The highest number of Dengue positives were detected in Epi Week 29.
(TIF)

S1 File Dataset for analysis (descriptive and graph). (XLSX)
