# Supplementary figures and images for: Outbreak of dengue fever in Ghana: The emergence of DENV-1 serotype
Source: PLoS Negl Trop Dis. 2026 May 11;20(5):e0014248. doi: 10.1371/journal.pntd.0014248 (PMC13215617; doi:10.1371/journal.pntd.0014248)

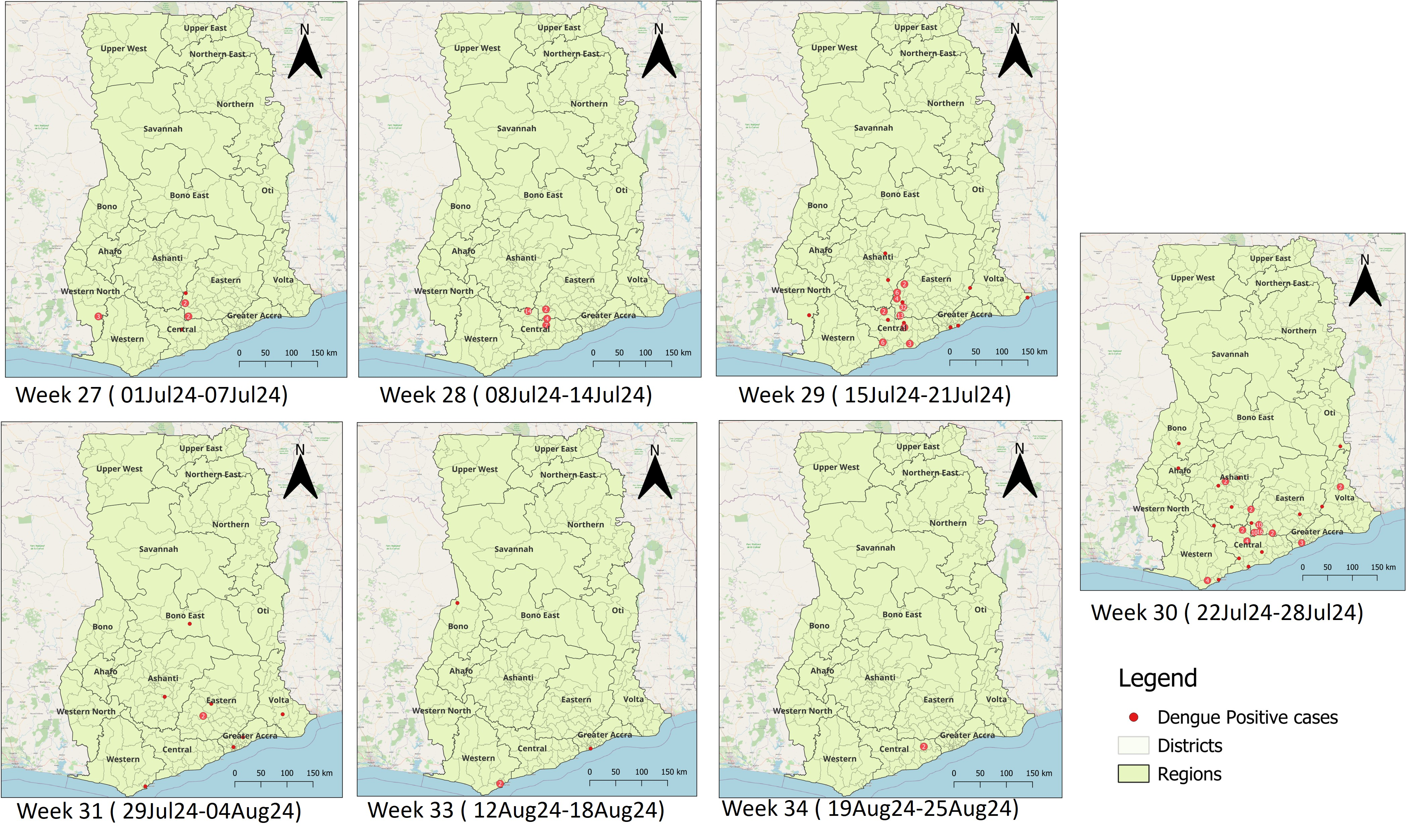

Supplement: S1 Fig — The map was created using QGIS v3.26.1-Buenos Aires. The administrative boundaries coordinates obtained from Ghana COD-AB (Humanitarian Data Exchange, https://data.humdata.org/dataset/cod-ab-gha), CC BY 4.0. Basemap: OpenStreetMap “Standard” (OpenStreetMap contributors; data under ODbL 1.0, https://www.openstreetmap.org/copyright). The red dots represent a single DENV positive within the districts and regions of Ghana. A red dot with a number in the middle symbolized the number dengue cases detected with that area. The highest number of Dengue positives were detected in Epi Week 29. (TIF) [file pntd.0014248.s001.tif]
